# BK_Ca_ (*Slo*) Channel Regulates Mitochondrial Function and Lifespan in *Drosophila melanogaster*

**DOI:** 10.3390/cells8090945

**Published:** 2019-08-21

**Authors:** Shubha Gururaja Rao, Piotr Bednarczyk, Atif Towheed, Kajol Shah, Priyanka Karekar, Devasena Ponnalagu, Haley N. Jensen, Sankar Addya, Beverly A.S. Reyes, Elisabeth J. Van Bockstaele, Adam Szewczyk, Douglas C. Wallace, Harpreet Singh

**Affiliations:** 1 Department of Physiology and Cell Biology, The Ohio State University Wexner Medical Center, Columbus, OH 43210, USA; 2 Department of Pharmacology and Physiology, Drexel University College of Medicine, Philadelphia, PA 19102, USA; 3 Department of Biophysics, Warsaw University of Life Sciences- SGGW, 02-776 Warsaw, Poland; 4 Center for Mitochondrial and Epigenomic Medicine, The Children’s Hospital of Philadelphia, Philadelphia, PA 19104, USA; 5 Kimmel Cancer Centre, Thomas Jefferson University, Philadelphia, PA 19107, USA; 6 Laboratory of Intracellular Ion Channels, Nencki Institute of Experimental Biology, 02-093 Warsaw, Poland; 7 Department of Pathology and Laboratory Medicine, University of Pennsylvania, Philadelphia, PA 19104, USA

**Keywords:** potassium channel, mitochondria, reactive oxygen species, antioxidants, life span, aging, BK_Ca_ channels

## Abstract

BK_Ca_ channels, originally discovered in *Drosophila melanogaster* as *slowpoke* (*slo*), are recognized for their roles in cellular and organ physiology. Pharmacological approaches implicated BK_Ca_ channels in cellular and organ protection possibly for their ability to modulate mitochondrial function. However, the direct role of BK_Ca_ channels in regulating mitochondrial structure and function is not deciphered. Here, we demonstrate that BK_Ca_ channels are present in fly mitochondria, and *slo* mutants show structural and functional defects in mitochondria. *slo* mutants display an increase in reactive oxygen species and the modulation of ROS affected their survival. We also found that the absence of BK_Ca_ channels reduced the lifespan of *Drosophila*, and overexpression of human BK_Ca_ channels in flies extends life span in males. Our study establishes the presence of BK_Ca_ channels in mitochondria of *Drosophila* and ascertains its novel physiological role in regulating mitochondrial structural and functional integrity, and lifespan.

## 1. Introduction

The large-conductance potassium channel activated by calcium (Ca^2+^) and voltage (BK_Ca_/Slo/MaxiK) was originally cloned in *Drosophila* at the *slowpoke (slo)* locus [1,2,3] and addressed as *Kcnma1* in mammals. BK_Ca_ channel is ubiquitously present in the plasma membrane of all eukaryotic cells. In *Drosophila,* extensive work has been performed on *slo* mutants where BK_Ca_ was shown to carry transient Ca^2+^-dependent K^+^ currents (I_KCa_) in muscles [2,4], and neuronal cells [5]. In addition, *slo* mutant has revealed roles of BK_Ca_ channel in neuronal functions, abnormal circadian activity, and well-characterized locomotor disorder (hence the name *slowpoke*) [3,6].

In mammals, BK_Ca_ is characterized to play similar roles in neuronal and non-neuronal cells [7]. They are the key ion channels with a large conductance, activated by gasses and lipids in addition to sensing changes in Ca^2+^, and voltage. In the last decade, mutations in *Kcnma1* gene have been associated with a paroxysmal movement disorder, epilepsy, obesity, hypertension, and cancer in humans [8]. BK_Ca_ null mutant mice showed alterations in circadian rhythm, blood pressure, hearing, heart rate, bladder control, locomotion, reproductive function, neurovascular coupling, airway constriction, insulin secretion, and learning and memory [7,9]. In the absence of BK_Ca_, the survival of mice and weight gain was hampered [10] but in contrast, the absence of Slo-1 in *Caenorhabditis elegans* was associated with slow motor aging and moderate extension of life span [11]. The majority of these functions were shown to be associated with the BK_Ca_ localized to the plasma membrane [9]. One exception to plasma membrane localization of BK_Ca_ channels is their localization to mitochondria of murine and rodent adult cardiomyocytes [8,12]. In the heart, activation of BK_Ca_ is known to play a direct role in cardioprotection from ischemia-reperfusion (IR) injury possibly via regulation of mitochondrial function [8,12,13,14].

Mitochondria are energy-generating organelles of the cell involved in several metabolic and signaling pathways. The inner mitochondrial membranes support the electron transport chain (ETC) tightly-coupled with membrane potential (ψ_mito_) that participates in the generation of ATP. Defects in ETC, ψ_mito_, mitochondrial fusion–fission events, or ionic imbalance can cause mitochondrial permeability transition pore (mPTP) to form, and result in apoptosis [15]. One of the well-established consequences of mitochondrial dysfunction is life span [16]. Several ion channels present in the plasma membrane and intracellular organelle membranes are known to regulate mitochondrial structure as well as functional integrity [8]. Even though BK_Ca_ is shown to regulate mitochondrial function, there is no direct evidence that BK_Ca_ can directly regulate mitochondrial structural and functional integrity. Expression of BK_Ca_ in coronary arteries from old rats, as well as humans, diminishes without showing any changes in biophysical properties [17]. However, whether BK_Ca_ directly affects life span is not well studied. To address this question, we studied the BK_Ca_ channel mutant (*slo*) phenotypes with respect to mitochondrial functional integrity and life span using the *Drosophila* model.

In this study, we found that BK_Ca_/Slo is present in mitochondria of *Drosophila* as a functional ion channel. The absence of BK_Ca_ results in age-related changes in mitochondrial structural and functional integrity. We also tested whether increased mitochondrial reactive oxygen species (ROS) is responsible for the early death of flies and chelating ROS could partially rescue the aging phenotype. Ablation of BK_Ca_ dramatically reduced the lifespan of *Drosophila*, while overexpression of human BK_Ca_ form surprisingly increased lifespan only in males. In agreement, our microarray data revealed various life span regulated transcripts altered in *slo* mutant flies. Taken together, our results define a novel function for BK_Ca_ channel in regulating mitochondrial structure and function and reduction in life span.

## 2. Materials and Methods

### 2.1. *Drosophila* Stocks, Reagents, Dyes, and Antibodies

All fly stocks were maintained at 25 °C on standard medium (jazz mix, nipagin free) unless otherwise stated. The experiments were carried out at 25 °C or 29 °C (for Gal4 efficiency) as mentioned in the results sections or figures. The Canton S strain served as the wild-type (wt) stock and is indicated as ‘wt’ through the manuscript. The *slo*^1^ mutants (chemical-induced mutation, originally characterized in Elkins et al. 1986 [3]), RNAi lines, Gal4 lines, and wild type lines (Canton S and W1118) were obtained from the Bloomington Stock Center. UAS Sod2 flies were a gift from Prof. David Walker (UCLA).

### 2.2. Immuno Cyto/Organelle Chemistry

Flight muscles were dissected and fixed with 4% (*w/v*) paraformaldehyde (PFA), washed and permeabilized with 0.4% (*v/v*) Triton-X100. Mitochondria were isolated from whole flies and loaded with mitotracker as described earlier [18]. Mitochondria and tissues were blocked with normal goat serum (10%) and stained with primary antibodies (anti-ubiquitin 1:100 (FK2), and anti-BK_Ca_ 1:200) and secondary antibodies, followed by DAPI (for tissues) (n = 5 independent experiments).

### 2.3. Electrophysiology

Patch-clamp experiments using mitoplasts (mitochondria without outer membranes) were performed as described previously [19,20]. Briefly, mitoplasts were prepared from mitochondria isolated from whole *D. melanogaster* placed in a hypotonic solution (5 mM HEPES, 100 μM CaCl_2_, pH = 7.2) to induce swelling and eventual disruption of the outer membrane. To restore the sample to an isotonic condition (150 mM KCl, 10 mM HEPES, 100 μM CaCl_2_, pH 7.2) a hypertonic solution (750 mM KCl, 30 mM HEPES, 100 μM CaCl_2_, pH 7.2) was added. The patch-clamp pipette was filled with an isotonic solution. Mitoplasts are easily recognizable due to their size, round shape, transparency, and the presence of a ‘cap’, characteristics that distinguish these structures from the cellular debris that is also present in the preparation. The low-calcium solution (1 μM CaCl_2_) contained the following: 150 mM KCl, 10 mM HEPES, 1 mM EGTA, and 0.752 mM CaCl_2_ at pH 7.2. An isotonic solution containing 100 μM CaCl_2_ was used as the control solution for all of the presented data. The experiments to assess the channel activity were carried out in patch-clamp inside-out mode [20]. The electrical circuit was made using Ag/AgCl electrodes and an agar salt bridge (3 M KCl) as the ground electrode. The current was recorded using a patch-clamp amplifier Axopatch 200B. The pipettes had a resistance of about 14 MΩ and were pulled using a vertical puller.

**Figure 1:**
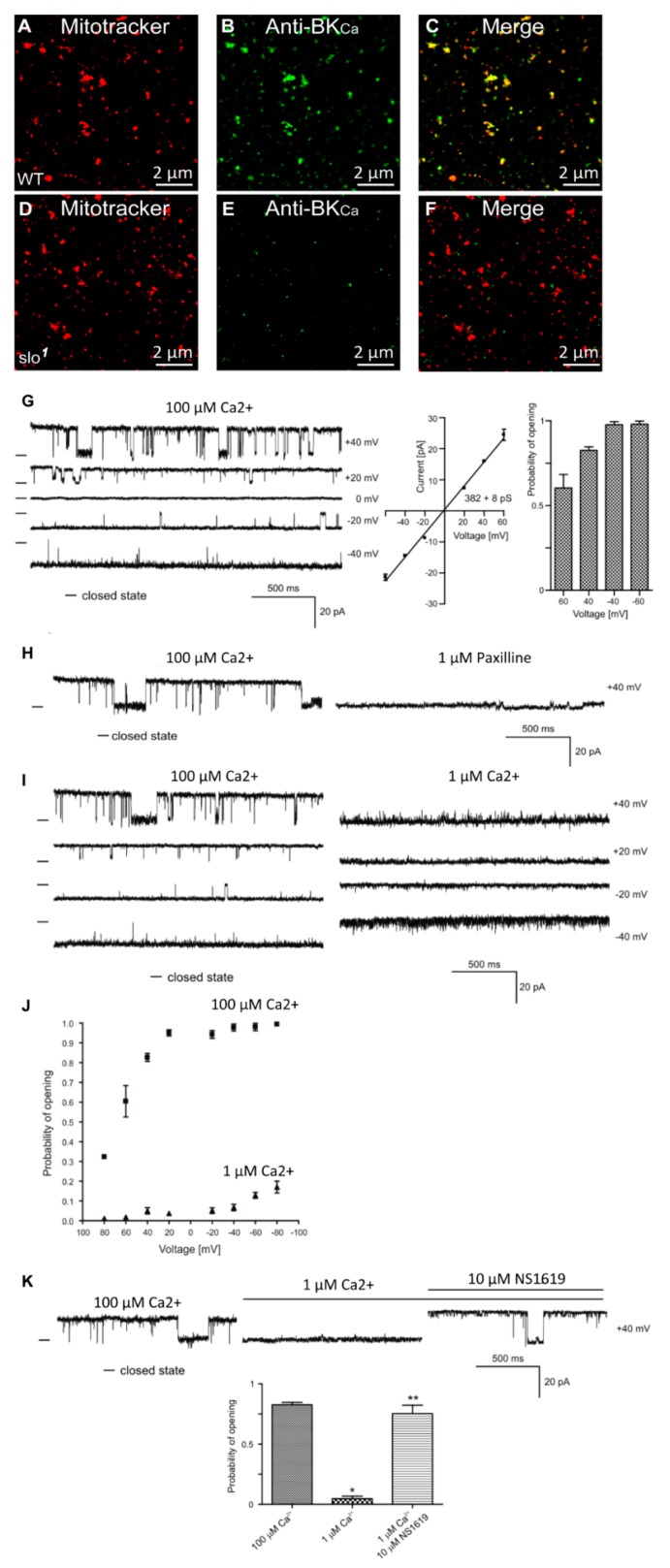
Localization of dSlo in isolated mitochondria. High-resolution confocal images of isolated mitochondria from *Drosophila* (**A**–**C**, wild type, **D**–**F** *slo* mutants) loaded with mitotracker (**A**,**D** red) and labeled with an anti-Slo antibody (**B**,**E** green). Overlays are shown in (**C**,**F**). Protein proximity index for dSlo to mitotracker was 0.5 ± 0.1. (**G**), Single-channel current-time recordings (left panel), current-voltage characteristics (middle panel) and Po analysis of single-channel events in a symmetric 150/150 mM KCl isotonic solution (100 μM Ca^2+^) at different voltages in mitoplast prepared from whole flies. (**H**), Effects of 1 μM Paxilline on the single-channel activity. (**I**), Single-channel current-time recordings in symmetric 150/150 mM KCl isotonic solution at control (100 μM Ca^2+^) and after reduction calcium concentration to 1 μM Ca^2+^. (**J**), Analysis opening probability in the presence of 1 and 100 μM Ca^2+^ at different voltages of the mitoBK_Ca_ channel in mitoplast prepared from whole flies. All data were acquired in a symmetric 150/150 mM KCl isotonic solution (n = 4). (**K**), Current–time recordings of single-channel activity in symmetric 150/150 mM KCl isotonic solution at control (100 μM Ca^2+^), after reduction calcium concentration to 1 μM Ca^2+^ and after application of 10 μM NS1619. The bar graph shows the distribution of the Po under the conditions above. * *p* < 0.001 vs. the control. ** *p* < 0.001 vs. 1 μM Ca^2+^. The data in (**G**,**J**,**K**) are presented as the means ± S.D. The recordings were low-pass filtered at 1 kHz. “-“ indicate a closed state of the channel.

The currents were low-pass filtered at 1 kHz and sampled at a frequency of 100 kHz. The traces of the experiments were recorded in single-channel mode. The conductance of the channel was calculated from the current–voltage relationship (Figure 1I). The probability of channel opening (Po, open probability) was determined using the single-channel search mode of the Clampfit software. Data from the experiments are reported as the mean values + standard deviations (S.D.). Student’s *t*-test was used for statistical analysis (n = 5 independent experiments comprising of mitochondrial isolation from 100 flies each).

### 2.4. Reactive Oxygen Species and Quantification

#### 2.4.1. Dihydroethidium (DHE)

Flight muscles were dissected quickly and placed in DHE (molecular probes) in PBS (1:1000 dilution) for 3 min and then washed in PBS three times for 3 min each. The samples were then fixed in 4% (*w/v*) PFA for 3 min and then washed again in PBS twice for 2 min each time. Flight muscles were then mounted in PBS and immediately photographed under a Zeiss confocal microscope (n = 5 independent experiments).

#### 2.4.2. Spectrophotometric Analysis

Flies were homogenized using a pestle, and ROS generation was detected from isolated mitochondria by amplex red using fluorescence spectrophotometer (Hitachi F-2710) described previously [18]. Briefly, 5 μg horseradish peroxidase (Sigma-Aldrich, St. Louis, MO, USA) was added to the ROS buffer (mmol/L, 20 Tris-HCl, 250 sucrose, 1 EGTA-Na_4_, 1 EDTA-Na_2_, pH 7.4 at 37 °C) and the baseline fluorescence was obtained (excitation at 560 nm and emission at 590 nm) for 30 min (n = 4 independent experiments with 100 flies each to isolate mitochondria). The protein concentration was used to normalize the amount of mitochondria from the same extracts.

### 2.5. ATP Measurement

ATP was measured from five individual groups of 2-week old females flies (n = 5 independent experiments with 20 flies each), using Roche ATP bioluminescence assay kit CLS II according to manufacturer’s instructions. Briefly, flies were homogenized in the lysis buffer and incubated for 5 min at room temperature. The extract was spun down at 10,000 *g* and the supernatant was transferred into a microwell plate. Upon addition of luciferase reagent, the luminescence was measured using a luminometer. The ATP measurements were normalized to protein from the same extract.

### 2.6. Oxygraph

Mitochondria from 40 flies were harvested and resuspended in 100 μL MiRO5 buffer (mmol/L, 0.5 EGTA, 3 MgCl_2_, 60 K-lactobionate, 20 taurine, 10 KH_2_PO_4_, 20 HEPES, 110 sucrose and 1 g/L BSA essentially fatty acid-free adjusted to pH 7.1). The assay was performed using the OROBOROS^®^ Oxygraph-2k (O2k, Oroboros Instruments, Innsbruck, Austria) similar to previously published methods [21]. The oxygen electrodes were calibrated with air-saturated respiration medium (MiRO5) at 25 °C as per manufacturer instructions. SUIT protocol was used to test the activities of Complex I (malate and pyruvate) and Complex II (rotenone and succinate). Following substrates and inhibitors were added sequentially: malate (2 mM) and pyruvate (5 mM), succinate (10 mM), rotenone (0.5 μM), malonic acid (5 mM), and antimycin A (2.5 μM). ADP (1–5 mM) was added at distinct steps after the addition of Complex I and II substrates. FCCP (Carbonyl cyanide 4-(trifluoromethoxy)phenylhydrazone) titrations (0.05 μM steps) were carefully performed to obtain maximum electron transport capacity. Cytochrome c (10 μM) test was performed in each experiment to make sure that the mitochondrial membrane integrity was not compromised. The rate of oxygen consumption (oxygen flux) as a function of time was normalized to the total protein concentration for each experiment. Background calibration and air calibration were performed as suggested by the manufacturer prior to the experiments. Data were analyzed by DatLab software (v5.0, Oroboros Instruments, Innsbruck, Austria). n = 5 independent experiments from 40 flies each. The protein content was used to normalize the amount of mitochondria from the same extracts.

### 2.7. Electron Microscopy

*Drosophila* flight muscles were dissected in PBS and fixed in 2% (*w/v*) glutaraldehyde and 2.5% (*w/v*) formaldehyde in PBS. Embedding, thin sectioning, and staining were carried out according to a standard protocol [22] (n = 5 independent preparations).

### 2.8. *Drosophila* Survivorship (Life Span Assays)

Flies were collected upon eclosure and reared in vials (30 flies in each vial, n = 3 independent assays) with food at 25 °C or 29 °C on a standard medium. The media was changed every 3 days and a number of deaths were recorded until all the flies died (for every life span assay, we used at least n ≥ 20 flies per vial and 3 such vials as biological replicates).

### 2.9. *Drosophila* Geotaxis

A negative geotaxis assay [23] was performed by counting the number of flies that cross 5 cm mark in 18 s after tapping them to the bottom of the vial (n = 3 independent experiments with 5 flies in each experiment).

### 2.10. Dye-Feeding Assay

Flies were fed fluorescein (2% (*w/v*) in media) dye for 9 h and imaged under a fluorescent microscope using 10X objective (Zeiss) (n = 5 independent experiments).

### 2.11. Paraquat/Glutathione Assays

Flies (2–3 days old) were starved for 2 h in 0.5% (*w/v*) agar and transferred into vials containing fiberglass filter papers with 5% (*w/v*) sucrose and 20 mM PQ with or without 220 µM glutathione (reduced). For the glutathione experiments, just eclosed flies were also reared on media containing 220 µM glutathione (reduced) for 1 week prior to PQ survival studies. Numbers of dead flies were counted every 12 h and plotted (n ≥ 20).

### 2.12. Cloning of Human BK_Ca_/UAS-Flies

BK_Ca_-HF full length was cloned in pUASTattb vector between NotI and XhoI restriction enzyme sites. The construct was amplified by PCR using N-terminal c-myc tag pCDNA3BK_Ca_-HF as a template. Briefly, BK_Ca_-HF was amplified using sense primer 5′-AAG GAA AAA AGC GGC CGC ATG GGC GCC GAG GAG CAG AAG-3′ and anti-sense primer 5′-CTA GTC TAG ACT CGA GTC AAA GCC GCT CTT CCT G-3′. The PCR conditions were 95 °C for 5 min, 30 cycles of 95 °C for 30 s, 55 °C for 30 s and 72 °C for 7 min followed by extension at 72 °C for 10 min. The clones obtained were confirmed by sequencing (Genewiz). Constructs were injected into *Drosophila* embryos using services from BestGene Inc (Chino Hills, CA, USA).

### 2.13. Microarray

Total RNA was isolated from 3-week-old female wild type and *slo* mutant flies using Qiagen RNAeasy kit. RNA was treated with RNase-free DNAse I. RNA was quantified on a Nanodrop ND-100 spectrophotometer (NanoDrop Technologies, Wilmington, DE, USA), followed by RNA quality assessment by analysis on an Agilent 2100 bioanalyzer (Agilent, Palo Alto, CA, USA). Fragmented biotin-labeled cDNA (from 100 ng RNA) were prepared using the GeneChip WT Plus kit.

Each Affymetrix gene chip *Drosophila* array (Affymetrix, Santa Clara, CA, USA) was hybridized with the fragmented and biotin-labeled target (4.5 μg) in 200 μL of hybridization cocktail. Target denaturation was performed at 99 °C for 2 min and then 45 °C for 5 min, followed by hybridization for 18 h. Then the arrays were washed and stained using GeneChip Fluidic Station 450, and hybridization signals were amplified using antibody amplification with goat IgG (Sigma-Aldrich, St. Louis, MO, USA) and anti-streptavidin biotinylated antibody (Vector Laboratories, Burlingame, CA, USA). The chips were scanned on an Affymetrix Gene Chip Scanner 3000, using Command Console Software. Background correction and normalization were done using Robust Multichip Average with Genespring V 14.9 software (Agilent). A 1.5-fold differentially expressed gene (*p* ≤ 0.05 values) list was generated. The listing of differentially expressed genes and their fold change were loaded into Ingenuity Pathway Analysis (IPA) 5.0 software (Qiagen Inc., https://www.qiagenbioinformatics.com/products/ingenuity-pathway-analysis) to perform biological network and functional analyses. IPA converts gene sets (with or without expression information) into related molecular networks based on IPA knowledge database. Core analysis was performed for and the genes were categorized based on molecular function, mapped to genetic networks, and ranked by score. The score reflects the probability that a collection of genes equal to or greater than a number in the network could not be achieved by chance alone. A score of more than 10 was used as a cutoff for identifying specific gene networks (n = 3 independent experiments with RNA isolated from 100 flies each).

### 2.14. Data Analysis

Data were analyzed using Sigma plot. Student’s *t*-tests or ANOVA were used for analyzing all the data and reported as mean + standard error or the mean in text. *p*-values less than 0.05 were considered significant.

## 3. Results

### 3.1. Presence of BK_Ca_ Currents in the *Drosophila* Mitochondria

In addition to the plasma membrane, BK_Ca_ channels are known to be present in the mitochondria of rodent neurons [24] and endothelial cells [19]. In adult cardiomyocytes, they are exclusively present in the mitochondria [12,25] but not in the plasma membrane [12,26]. In *Drosophila*, BK_Ca_ has been well-characterized in the plasma membrane at the biophysical and physiological levels [7,27], however, it is not known whether it is present or active in the mitochondria. In order to test for the presence of BK_Ca_ in mitochondria, we loaded isolated mitochondria with mitotracker [18] and labeled with anti-BK_Ca_ antibodies (Figure 1A,B,D,E). Mitochondria isolated from the whole wild-type but not *slo*^1^ mutant flies [1,3] showed the presence of a BK_Ca_-specific signal (Figure 1A–F). Protein proximity index (PPI) analysis [12] to estimate colocalization of BK_Ca_ to mitotracker-loaded mitochondria showed a value of ~0.5 ± 0.1 (n = 6), indicating ~50% of BK_Ca_ signal colocalized with mitochondria.

BK_Ca_ has been recorded from cardiac and endothelial mitoplasts [8,19,25], but not in *Drosophila* mitoplast (inner membrane of mitochondria). To examine whether BK_Ca_ is active in *Drosophila* mitoplast (n = 5 independent experiments, mitochondria isolated from 100 flies each), we isolated mitoplast from wild type flies and carried out patch-clamp analysis [19]. Approximately 80% of the currents detected in the mitoplasts were attributed to BK_Ca_-specific channels. We recorded channel activity (Figure 1G) in the presence of 100 μM Ca^2+^ in the bath pipette at holding potentials ranging from +60 mV to −60 mV in a symmetrical solution (150 mM KCl, 10 mM HEPES, 100 µM Ca^2+^, pH 7.2). The current (I) vs. voltage (V) curve (Figure 1G) calculated from the single-channel currents showed a conductance of 382 ± 8 pS (n = 5) for mitoBK_Ca_. Surprisingly, the open probability of single-channel current increases from ~0.6 at +60 mV to ~1.0 at −60 mV holding potentials (Figure 1G). On addition of paxilline (BK_Ca_ antagonist), the large channel conductance was completely blocked (Figure 1H), confirming that the large currents were originated from paxilline-sensitive BK_Ca_.

Since BK_Ca_ is a Ca^2+^-sensitive channel, we also changed the Ca^2+^ concentration of bath solution from 100 μM to 1 μM. Single-channel recordings showed a decrease in open probability (Po) at holding potentials ranging from −40 mV to 40 mV (Figure 1I). Po vs. V plot shows increase in Po at 100 μM as compared to 1 μM Ca^2+^ (Figure 1J). Large conductance channels were not observed at 1 μM Ca^2+^. However, on the addition of 1 μM NS1619 (BK_Ca_ agonist), the large-conductance channel reappeared with a high Po (Figure 1K). Our immuno-organelle chemistry data indicate the presence of BK_Ca_ channels in isolated mitochondria. In addition, our electrophysiological approach demonstrates the presence of BK_Ca_ in *Drosophila* mitochondria corroborating the immuno-organelle chemistry data.

### 3.2. Mitochondrial Functional Aberrations in BK_Ca_ Mutants

Given the presence of BK_Ca_ in the mitochondria, we sought to investigate if BK_Ca_ plays a direct role in its functional integrity using the BK_Ca_ (*slo*^1^) mutant [1,28].

**Figure 2:**
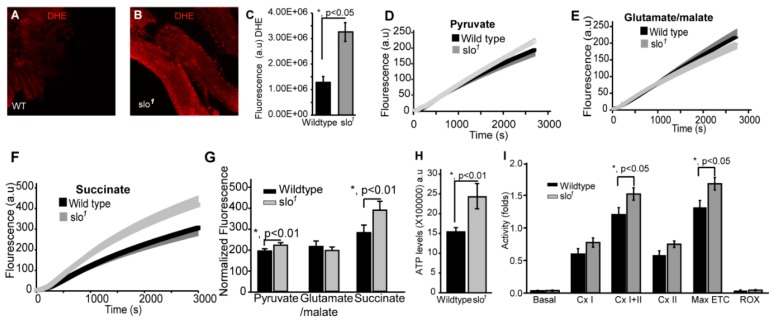
Mitochondrial functional defects in *slo*^1^ mutants. (**A**) (wt) and (**B**) (*slo*^1^) show indirect flight muscles stained with DHE to detect ROS. (**C**) Quantification of ROS fluorescence in (**A**) and (**B**) (wt-black, *slo*^1^-grey). The graphs in (**D**–**F**) show ROS generation in isolated wt (black) and *slo*^1^ mutant mitochondria (gray) in the presence of pyruvate (**D**), glutamate/malate (**E**), or succinate (**F**) as substrates. Succinate and pyruvate, but not glutamate/malate show an increase in ROS as detected by the amplex red dye, compared to wt mitochondria. (**G**) Quantification of (**D**,**E**), and (**F**,**H**) shows ATP levels increased in *slo*^1^ mutants (wt-black, *slo*^1^-grey). (**I**) Quantification of oxygen flux from enriched mitochondria from 40 thoraces of wt (black) and *slo*^1^ mutants (gray) flies. Basal, complex I, complex II, and ROX (non-mitochondrial residual oxygen consumption rate) oxygen consumption rates do not significantly vary between wt and *slo*^1^ mutants but combined complex I and complex II and maximum ETC consumption are significantly higher in *slo*^1^ mutants.

We tested if ROS, the major byproduct of mitochondria, is altered in *slo*^1^ mutants. The *slo*^1^ mutant showed higher levels of DHE staining (a detector of ROS) in indirect flight muscles indicating significantly (*p* < 0.05, n = 5) increased production and accumulation of ROS (Figure 2A vs. 2B, quantified in 2C). We examined ETC function where ROS is generated and found that *slo*^1^ mutant mitochondria showed a significant increase in ROS production (Figure 2D–G). The increase was significant when pyruvate was used as a substrate (Figure 2D,G, *p* < 0.01). To dissect which complex is generating this ROS, we used specific substrates for complex I and complex II of ETC. With glutamate/malate (substrate for complex I), we did not see any significant difference in ROS generation (Figure 2E,G). However, succinate (substrate for complex II) showed a much higher level of ROS generation (Figure 2F,G) in *slo*^1^ mutants, indicating that increased ROS produced could be due to complex III and or backflow of electrons to complex I [29]. Another mutant for dSlo, *slo[f05915]* [30] also showed the elevated rate as well as the amount of ROS production by complex III (Supplementary Figure S1).

During oxidative phosphorylation, the energy released from oxidation/reduction reactions drives the synthesis of ATP. Mitochondrial disintegration is often associated with a decrease in ATP-generation [31]. We tested ATP-generation by mitochondria from two-week-old wt and *slo*^1^ mutant flies. Surprisingly, *slo*^1^ mutant flies showed a significant increase (*p* < 0.001, n = 5) in ATP-generation compared to wt flies (Figure 2H). We also measured the activity of complexes from both wt and *slo*^1^ mutant flies by measuring substrate driven oxygen consumption rates. In comparison to wt, *slo*^1^ mutant flies had similar basal rates and higher but not significant complex I and complex II oxygen consumption rates (Figure 2I). However, upon substrate saturation of both complex I and II combined, the oxygen consumption rate was highly significant (*p* < 0.05, n = 5, Figure 2I). The maximum electron transport system (ETS) capacity was also increased in *slo*^1^ mutants suggesting a higher index of mitochondrial uncoupling in these mutant mitochondria (Figure 2I). We did not observe a sex-based difference between in *slo*^1^ mutants.

### 3.3. Absence of BK_Ca_ Renders Flies Susceptible to Oxidative Stress

**Figure 3:**
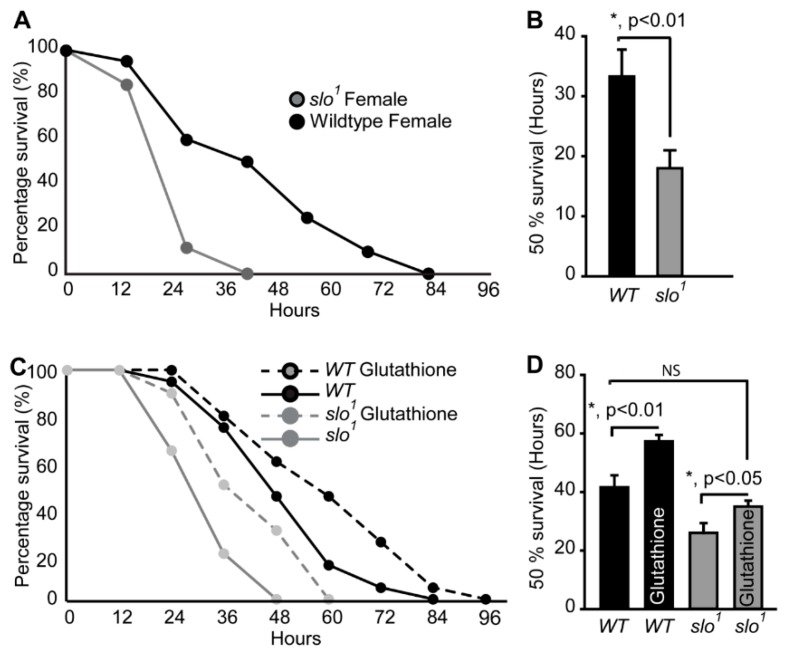
Oxidative stress on fly survival. (**A**) Survival of *slo*^1^ mutants is significantly lower compared to wt flies fed on 20 mM PQ in 5% (*w/v*) sucrose. (**B**) Histogram shows 50% survival for and *slo*^1^ and wt flies. (**C**) Survival of *slo*^1^ mutants while PQ feeding with or without glutathione. (**D**) Histogram shows 50% survival for and *slo*^1^ mutants with or without reduced glutathione (GSH). GSH increased the survival of wild-type and *slo*^1^ mutants.

Our findings indicate abnormally hyper-functional mitochondria, which explain the higher level of ROS production from the mitochondria. To analyze if increased ROS renders *slo*^1^ mutant flies sensitive to oxidative stress, we fed them with paraquat (PQ), a compound known to induce oxidative stress [32]. We found that *slo*^1^ mutants (Figure 3A) are highly sensitive to PQ feeding. Flies (2–3 days old) maintained on starvation media for 2 h followed by exposure to 5% (*w/v*) sucrose combined with 20 mM PQ showed 50% death of *slo*^1^ flies within 15 h whereas the 50% wt survived up to 25 h (Figure 3A,B). Hypersensitivity of *slo*^1^ mutants to PQ was highly intriguing indicating that ROS plays a detrimental role on the survivability of *slo*^1^ mutants. We tested hypersensitivity to ROS by feeding the flies with reduced glutathione (GSH) to see if glutathione feeding helps them survive in oxidative stress. We observed improved survival of *slo*^1^ mutants similar to wild type in PQ treatment upon feeding of GSH (Figure 3C,D). These results show that increased ROS in *slo*^1^ mutants is responsible for oxidative damage and perhaps influences the survival of flies.

### 3.4. Mitochondrial Structural Abnormalities in BK_Ca_ Mutants

**Figure 4:**
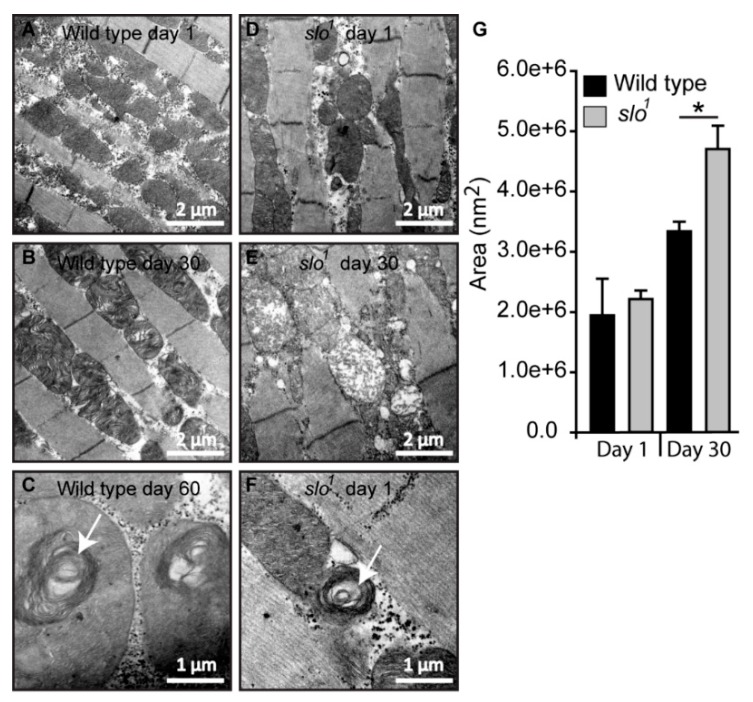
Mitochondrial structural defects in *slo*^1^ mutants. (**A**) Wt mitochondria from indirect flight muscles at age 1 day show normal cristae organization. The *slo^1^* mutant mitochondria also show normal structure but there are increased numbers of vacuoles in the muscles (**D**). (**B**) Wt mitochondria at the age of day 30 also show a normal cristae organization. However, the *slo^1^* mutant mitochondria show disorganized cristae and swollen mitochondria (**E**). (**C**) Mitochondria of very old flies (at day 60) in wt show swirling of cristae, a phenotype characteristic of old age but occasionally young (day 1) *slo^1^* flies also show such swirls (**F**), indicated by white arrows. (**G**) The Average area indicated by histogram showed no difference in day 1 mitochondria in between wt (black) and *slo^1^* (gray) but significant (* *p* < 0.05) difference at day 30.

In order to study the structure of mitochondria in *slo*^1^ mutant flies, we analyzed the ultrastructure of mitochondria in wt and *slo*^1^ flies (Figure 4, n = 5). Electron microscopic analysis revealed major differences in the ultrastructure of mitochondria (Figure 4B vs. E). We studied day 1 and day 30 time points based on the differences observed in our initial experiments. The number of mitochondria in *slo*^1^ mutant flies was less compared to wt from older flies (day 30). The mitochondria of older *slo*^1^ mutants showed severe defects in terms of cristae arrangement (Figure 4E). The size of mitochondria in *slo*^1^ mutant older flies was also increased as compared to the young flies, which could be attributed to their swollen appearance and loss of continuous inner mitochondrial membrane (Figure 4D,E,G). No major differences were observed between young (day 1) vs. older wt flies (day 30, Figure 4A,B,G).

Mitochondrial swirls are known to represent early events of deterioration. Unusually close packing of cristae in an onion peel arrangement in the flight muscle mitochondria makes it feasible to detect it by electron micrograph [33]. We observed sporadic mitochondrial swirls in very old wt flies (≥60 days, Figure 4C) but *slo*^1^ mutant showed mitochondrial swirls from day 1 in the flight muscle (Figure 4F, one to two occurrences per field). We have also observed the appearance of vacuoles in young *slo*^1^ mutant flies (Figure 4D) whereas they were not seen in the wt counterparts. Taken together these analyses indicate major disorganization in mitochondrial structure in *slo*^1^ mutant flies, some of them being hallmarks of the early aging phenotype. Mitochondrial structural disintegration, as well as the appearance of swirls, indicated possible oxidative damage to mitochondria consistent with our earlier results. Age-related abnormalities in mitochondria from flight muscles and other tissues of *Drosophila* are well-documented [34,35]. Older flies show severe mitochondrial deterioration; including loss of cristae, increase in size (swelling) and loss of arrangement in muscle fibers [33]. This prompted us to investigate if there are differences in the lifespan of *slo*^1^ mutant flies.

### 3.5. *slo*^1^ Mutants Show Reduced Lifespan

Mitochondria are energy-generating organelles of the cell involved in several metabolic and signaling pathways [15] such as lifespan. Our results showed mitochondrial structural and functional defects in BK_Ca_ mutants. Hence, we further investigated the consequence of absence of BK_Ca_ in lifespan.

**Figure 5:**
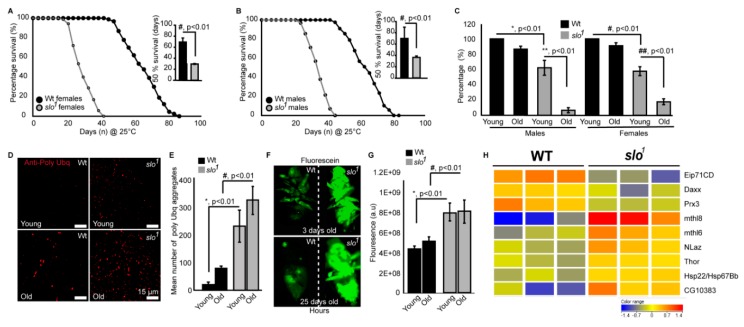
*slo*^1^ mutants reveal accelerated aging. *Drosophila* BK_Ca_ (*slo*^1^) mutants show significantly reduced lifespan of females (**A**) and males (**B**) by approximately 50% compared to wild-type (wt) flies. The inset shows 50% survival for wt (black) and *slo*^1^ (gray), which was reduced significantly for *slo*^1^ mutants. (**C**) Negative geotaxis assay for wt and slo flies at young (day 3) and older flies (day 30) shows reduced ability of *slo*^1^ mutants to climb the marked distance in a given time in vials compared to their controls. (**D**) Increased polyubiquitination staining is observed in *slo*^1^ mutants (red) as compared to wt in both young and older ages and quantification is provided in (**E**). (**F**) *slo*^1^ mutants show increased intestinal perforations as determined by the leakage of fluorescein dye (green) from the gut unlike control flies, which only show the dye in their gut at both young and older ages. (**G**) Quantification of fluorescein signal from (**F**). (**H**) Microarray data showing differential expression of life span-related genes in wt and *slo^1^* mutants.

We compared the lifespan of *slo*^1^ mutants with wt flies. Even though flies were cultured in the optimal nutritional conditions and temperature (25 °C), *slo^1^* mutants surprisingly died within 45 ± 3 days (Figure 5A, B, and Supplementary Figure S2) whereas wt flies survived up to 85 ± 5 days, showing that the *slo*^1^ mutant has only ~50% of lifespan compared to wt flies. There was no significant difference between females (Figure 5A) and males (Figure 5B) *slo*^1^ mutants as they both showed decreased lifespan by ~50%. Reductions in the lifespan of female *Drosophila* are also associated with mating [36]. To test whether BK_Ca_ has any role in ‘cost of mating’, we performed a parallel study where males and females were housed together. We did not detect any significant differences in the observed lifespan of flies cultured separately or together (Supplementary Figure S2).

Age-related locomotor impairments including negative geotaxis [23] are well-documented in *Drosophila* [37]. *Drosophila slo*^1^ mutants are known to have locomotor impairments [38] which were also observed here in both males and females (Figure 5C). No significant changes in negative geotaxis were observed in wt flies in between 3 days and 30 days old in both genders. Wild type flies survive up to ~90 days and our geotaxis assays were performed on comparatively younger wild type flies. However, with age *slo*^1^ mutants showed a dramatic reduction in locomotion (Figure 5C, n = 3, 5 flies each in each trial). Reduction in lifespan is directly associated with increased proteotoxicity [39,40]. We characterized *slo*^1^ mutants at young and old age along with wt flies to study the age-related deposition of protein aggregates in a flight muscle by immunofluorescence (Figure 5D). As shown earlier [39,40] anti-Ubiquitin (Ubq) antibody labels’ protein aggregates in indirect flight muscle in old flies, we also observed a significant increase in protein aggregates (Figure 5D,E) in *slo*^1^ mutants. Surprisingly, the *slo*^1^ mutant showed a higher amount of aggregates from a young age which increased with old age (Figure 5D). Integrated fluorescence of protein aggregates showed a significant increase in Poly Ubq fluorescence with age in both wt and *slo*^1^ mutants (Figure 5E) (n = 5). We also observed similar results with western blot studies where poly-Ubq streak was increased in *slo*^1^ mutants. In corroboration, we observed an increase in the levels of refractory to Sigma P, Ref(2)P, a Drosophila orthologue of mammalian p62, which is a major component of protein aggregates in flies [41] (Supplementary Figure S3).

Age-dependent intestinal-perforations are utilized as markers of aging and physiological changes associated with aging [42]. We tested age-related intestinal perforation by feeding fluorescein dye to young and old flies from wt as well as *slo*^1^ mutant groups (Figure 5F). Surprisingly, the mutant flies showed fluorescent dye leakage through the intestinal perforations from a young age (3 days) indicating the premature or accelerated aging phenotype (Figure 5F). Taken together, our results suggest that *slo*^1^ mutants not only show shortened lifespan but several accelerated aging phenotypes (n = 5).

We conducted microarray studies using 3-week old wild type and *slo* mutants (n = 3) to investigate if life span related genes are differentially regulated in the mutants. We found several genes implicated in life span regulation altered in the *slo* mutants (Figure 5G). Methuselah mutants are well known to expand the life span of *Drosophila* [43]. We indeed found overexpression of two Methuselah genes explaining the converse phenotype of shortened life span in the *slo* mutants. Overexpression of methionine sulfoxide reductase A (Epi71CD) is shown to increase life span, whereas in our arrays we found a decrease of this enzyme, along with the mitochondrial antioxidant peroxiredoxin 3 [44]. Several other life span related genes were altered such as Thor, NLaz, Hsp22, and Daxx [45,46,47,48] suggesting the absence of BK_Ca_ channel having an important role in regulating life span. In line with the observed mitochondria-related oxidative stress in slo mutants, we also found 63 oxidative stress-related genes altered (Supplementary Figure S4).

**Figure 6:**
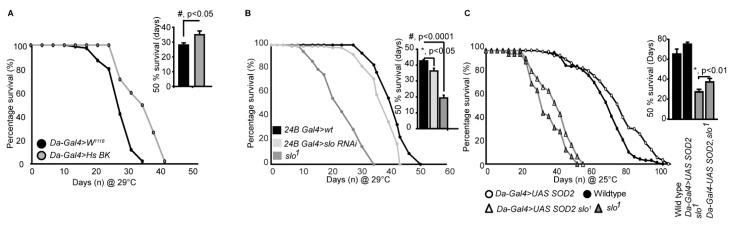
*slo*^1^ expression modulates survival. (**A**) Males overexpressing human (Hs) BK_Ca_ increase life span. Inset shows quantification of 50% survival of control and Hs BK_Ca_ overexpressing flies. (**B**) Lifespan of control, slo RNAi under 24B Gal4 and *slo*^1^ mutants at 29 °C. (**C**) *slo*^1^ mutants are partially rescued by the overexpression of Daughterless Gal4-UAS; Sod2. Inset histograms represent the 50% survivability of the mutants. Histograms represent the 50% survivability of the mutants in both (**B**) and (**C**).

We further wanted to investigate if overexpression of BK_Ca_ in flies has a converse effect on life span compared to the *slo* mutants. We created full-length BK_Ca_ pUAST plasmids and injected into flies. Consistent with our previous results in Figure 5A,B flies overexpressing human BK_Ca_ at 29 °C, at which Gal4 efficiency is maximum, resulted in an increase in a life span of male flies (Figure 6A). The effect was not seen in female flies where they had a similar life span compared to wild type flies (Supplementary Figure S5). This showed that BK_Ca_ has a definitive role in regulating life span and the function is genetically conserved.

Using RNAis against BK_Ca_ we also narrowed down that the reduction in lifespan is at least partly through its action in the muscles. We tested global (Daughterless Gal4), neuronal (Elav Gal4), and muscle (24B Gal4) knockdown of BK_Ca_ and found that muscle knockdown of BK_Ca_ showed a reduction in lifespan compared to control flies (Figure 6B). We found that *24BGal4>slo* RNAi decreased the lifespan (Figure 6B) from 52 ± 4 days to around 42 ± 3 days at 29 °C, at which Gal4 efficiency is maximum. The 50% survivability bar graphs show a significant decrease in the lifespan of *24BGal4>slo RNAi* (Figure 6B). The reduction in locomotor activity with age could also be associated with loss of BK_Ca_ in the muscles where mitochondria play an important role [49].

As ROS generation was elevated in *slo^1^* mutants, we attempted to rescue the reduction in lifespan of BK_Ca_ mutants by chelating ROS. We overexpressed SOD2 using UAS-SOD2 in *slo*^1^ mutants using a ubiquitous daughterless-Gal4 driver and cultured them to study their lifespan. As shown in Figure 6C, both wt, and *slo^1^* mutants showed a modest but significant increase in lifespan on overexpression of SOD2 at 25 °C (we observed similar results at 29 °C as well). We further calculated the time at which 50% of flies survived. Overexpression of SOD2 increased 50% survivability by 10% but for *slo^1^* mutants, we observed ~36 ± 8% increase. These results partially implicate ROS in the reduction of the life span of *slo^1^* mutants and chelating ROS rescued the lifespan of *slo*^1^ mutant flies. This suggests, in addition to ROS, other mitochondrial abnormalities observed in *slo*^1^ mutants could be contributing to the reduction of lifespan.

## 4. Discussion

The presence of BK_Ca_ in mitochondria has been extensively pursued in the mitochondrial channel field in recent years [8,50]. Hence, we investigated if *Drosophila* mitochondria contain BK_Ca_ currents. Our electrophysiological studies provide clear evidence for the presence of BK_Ca_ channels in the mitochondria of *Drosophila*. The currents measured are of typical BK_Ca_ characteristics and they could be blocked by paxilline, and activated by calcium. Surprisingly, Po decreased at higher voltages which needs further characterization as this phenomenon could result from the presence or absence of additional regulatory subunit. Our immunolabelling of mitochondria also confirmed localization of BK_Ca_ to the mitochondria. These experiments indicate that the large current in *Drosophila* mitoplast is highly sensitive to changes in Ca^2+^ concentrations in the mitochondrial matrix, and could be blocked by highly specific BK_Ca_ inhibitor, paxilline. The large-conductance and sensitivity to NS1619, paxilline as well as Ca^2+^ and voltage in addition to mitochondrial immunocytochemistry indicate that *Drosophila* mitoplast possess functional BK_Ca_ proteins. These experiments for the first time establish BK_Ca_ as a mitochondrial ion channel across the species confirming an evolutionary presence. However, BK_Ca_ is located in several other membranes, for example in plasma membranes in neurons and astrocytes [7,8], along with mitochondrial membranes. It is yet to be deciphered how mitochondrial function is controlled by BK_Ca_ with respect to its various locations.

The absence of BK_Ca_ has several consequences on mitochondria. A dramatic increase in the accumulation of ROS was observed in *slo*^1^ mutant flies. Increased ROS can be detected in live tissues of mutant flies and also a higher amount of ROS generation was observed in isolated mitochondria. Energized *slo*^1^ mutant mitochondria produce increased levels of ROS compared to wt mitochondria when provided with specific substrates. This indicates that the increased ROS is a consequence of dysfunctional mitochondria, although it does not rule out the contribution from NOX related enzymes that are capable of producing ROS. However, other mitochondrial readouts such as ATP and oxidative phosphorylation measurements further consolidate the hypothesis that the increased ROS observed in *slo*^1^ mutant animals is due to mitochondria. The increase in ATP could also be due to sustained membrane potential caused by reduced potassium leak, increasing proton flux from ATP synthase. These results indicate hyper-functional mitochondria, which explain the higher level of ROS production from the mitochondria. We have recently shown that genetic activation of BK_Ca_ channels reduces ROS upon IR injury stress [13], which further supports a role for BK_Ca_ in regulating ROS.

The BK_Ca_ flies also display oxidative stress sensitivity in a ROS-based pathway [51]. When we subjected the flies to oxidative stress using PQ, the BK_Ca_ mutant flies died within 48 h compared to wt flies, which survive for more than 3 days. This indicated that BK_Ca_ flies are under high oxidative stress and any further increase in ROS could be detrimental. The converse experiments by feeding glutathione increased the survivability of *slo*^1^ mutants indicating that ROS is at least a factor that determines the survival of *slo*^1^ mutants.

Consistent with the functional abnormalities of mitochondria, *slo*^1^ mutants also show several structural defects in mitochondria. Although younger flies contain mitochondria of normal appearance, occasional vacuoles and mitochondrial swirls are observed in flies even of day 1 age. As the flies age, mitochondrial structural defects are further enhanced where cristae structure is lost and mitochondrial swelling occurs. This depicts a progressive disintegration of mitochondria in an accelerated manner perhaps one of the causes leading to the early death of flies. Given that mitochondria from *slo*^1^ mutants produced higher amounts of ATP, it is possible that the absence of BK_Ca_ results in changes in cristae as observed here which results in assembly of respiratory chain supercomplexes (RCS). RCS are quaternary supramolecular structures that allow channeling of electrons amongst individual respiratory chain complexes facilitate selective use of RCC subsets for nicotine adenine dinucleotide (NADH)- or flavin adenine dinucleotide (FAD)-derived electrons [52]. These type of supramolecular organization is commonly found in cristae, and the mitochondrial ATP synthase is also assembled as dimers with increased ATPase activity and the dimerization is further augmented during autophagy [53]. Mitochondria are closely associated with lifespan and mitochondrial defects accumulate as the animal ages. Interestingly in *slo*^1^ mutants, mitochondrial abnormalities can be seen on day 1 or birth. Mitochondrial swirls are occasionally seen in *slo*^1^ mutants, a phenotype hallmark of very old/dying flies in wt situations.

Ion channels are reported to alter with age in rats and humans [17,54]. Expression of BK_Ca_ channels was shown to be reduced in aged coronary arteries possibly resulting in decreased vasodilator capacity, increased the risk of coronary spasm and myocardial ischemia in older people [17,54,55]. In mice, the absence of BK_Ca_ causes low body weight and decreased survivability in the first 10 weeks [10,56] but a complete life span analysis has not been reported. In contrast, a recent report indicated a moderate increase in life span and motor neuron activity in *C. elegans* BK_Ca_ mutants [11]. However, broad augmentation of endogenous BK currents in vivo (gain-of-function BK_Ca_ TG mice) resulted in protecting the heart from ischemia-reperfusion injury [13]. In our current study, we have discovered that *Drosophila* lacking BK_Ca_ showed a decrease in lifespan supporting mammalian observations. Flies mutant for BK_Ca_ not only die rapidly but show early and premature accumulation of aging markers. This indicates that the presence of BK_Ca_ is important in the regulation of aging. The key reason for this difference between *C. elegans*, flies, and mammals could be attributed to the role of electron transport chain (ETC) and ROS in aging. In *C. elegans* any perturbation with ETC results in an increase in life span due to their anaerobic energy-producing capacity, which is the exact opposite to what is observed in mammals and *Drosophila* [57]. One of the best examples for this difference is in frataxin homolog gene (frh-1), where knocking down frh-1 significantly increased the life span of *C. elegans* [58], but its ablation in mouse decreased life span [59], and recessive mutations in frataxin cause Friedreich’s ataxia [60] in humans.

In agreement with accelerated aging, we observed the accumulation of age-related phenotypes just after the birth of flies such as intestinal perforation and polyubiquitin aggregation accompanied by motor defects in *slo*^1^ mutant flies. This provides evidence that BK_Ca_ channel function is required from an early age, perhaps from developmental stages, for the animal to age in a wild type manner and it regulates life span. Our microarray data intriguing shows increased expression of several methuselah genes whose mutants are known to extend life span [43]. While it is not clearly shown if an increase in methuselah expression reduces the life span, it is consistent with the proposed role of methuselah where lack of it increases life span. We also observed several life span related genes altered in the *slo* mutants along with oxidative stress genes in our microarray [45,46,47,48]. These results collectively show that slo is a major regulator of oxidative stress and life span and a detailed study is required in the future to narrow down the direct role of BK_Ca_ in regulating life span. The major limitation is the contribution of mitochondrial vs. non-mitochondrial BKCa in regulation the lifespan of *Drosophila*.

Supporting our observation of ion channels regulating lifespan, it was recently shown that low temperatures activate a cold-sensitive cation channel TRPA-1, which extends a lifespan by triggering cellular signaling pathways [61]. It is also interesting that lack of BK_Ca_ only from the muscles also causes reduced lifespan similar to what is reported in earlier studies [62]. Conversely increasing BK_Ca_ by Gal4-UAS based overexpression increased the life span indicates a true role for BK_Ca_ in regulating life span. Human BK_Ca_ is 70% identical to *Drosophila* BK_Ca_ but is sufficient to rescues as well as augment the life span of *Drosophila* indicating the function could be conserved across species. This result is of relevance given expression of BK_Ca_ goes down with age in humans [63]. However, it is intriguing that only males show this effect while females do not show life span extension upon overexpression. These results are in agreement with increase in a life span of male flies on overexpression of specific DNA repair endonucleases [64]. DNA repair mechanisms are ATP-dependent processes, and dysfunctional mitochondria over a longer period of time could trigger apoptosis and cell death. This indicates gender-based differences in how BK_Ca_ regulates life span or could be involved in DNA repair mechanisms, which needs detailed study.

Taken together, our study establishes BK_Ca_/Slo as an important player in maintaining the structure and functional integrity of mitochondria in *Drosophila*, and regulating lifespan. These findings also corroborate earlier studies that expression of BK_Ca_ reduces during aging which increases the risk of cardiovascular diseases in older people [17]. Our study also proves the existence of ion channel activity for BK_Ca_ in the *Drosophila* mitochondria. Given the dual cellular localization (intracellular membranes vs. plasma membrane) of BK_Ca_, it is critical to evaluate its spatial specific role(s) in pathophysiology in future studies. In our findings, we have not ruled out the role of plasma membrane BK_Ca_ but introduced its new physiological role in aging. Presence of BK_Ca_ in the mitochondria and its role in modulation of ROS opens up avenues to explore antioxidant-based therapies in diseases and disorders related to these large conductance potassium channels. In the past decade, studies have indicated that pharmacological and genetic activation of BK_Ca_ results in cellular and organ protection from ischemic injuries. Despite recent successes with animal models, the translational aspect of BK_Ca_ channel openers is still lacking due to poor selectivity of these agonists. With recent advancements in gene delivery and gene therapy, our recent and current work reiterates the importance of expression of BK_Ca_ to protect organs from ischemic insult or increasing life span.

## Supplementary Materials

The following are available online at https://www.mdpi.com/2073-4409/8/9/945/s1, Figure S1: *slo^f05915^* mitochondria produce increased ROS. Figure S2: *slo*^1^ mutants result in accelerated aging when males and females are grown together. Figure S3: Increased levels of protein aggregates in *slo*^1^ mutants. Figure S4: Heat map of oxidative stress-related genes altered in *slo*^1^ mutants in comparison to wt flies. Figure S5: Females overexpressing human (Hs) BK_Ca_ do not show a change in life span as compared to control flies.
