# Supplementary material for: BKCa (Slo) Channel Regulates Mitochondrial Function and Lifespan in Drosophila melanogaster
Source: Cells. 2019 Aug 21;8(9):945. doi: 10.3390/cells8090945 (PMC6770356; doi:10.3390/cells8090945)

Supplementary Figure. S1

***slo*^{f05915} mitochondria produce increased ROS.** The graphs show isolated wild-type (black) and *slo*^{f05915} mitochondria (gray) with increased ROS produced by complex II/III, in the presence of succinate as substrate (**A**). Histograms show a significant increase in ROS in *slo*^{f05915} mitochondria (**B**).

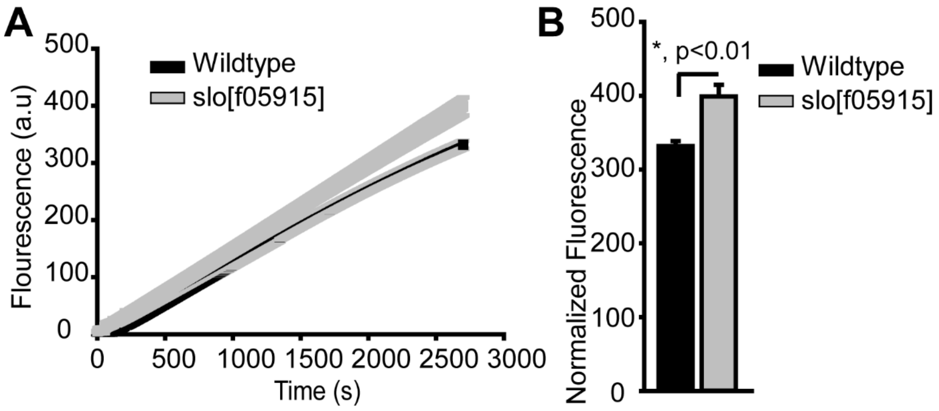

Supplementary Figure. S2

***slo*^{1} mutants result in accelerated aging when males and females are grown together.** *Drosophila* BK_{Ca} (*slo*^{1}) mutants show significantly reduced lifespan of males and females by approximately 50% compared to wt flies (A). The inset shows 50% survival for wt (black) and *slo*^{1} (gray), which was reduced significantly for *slo*^{1}(B).

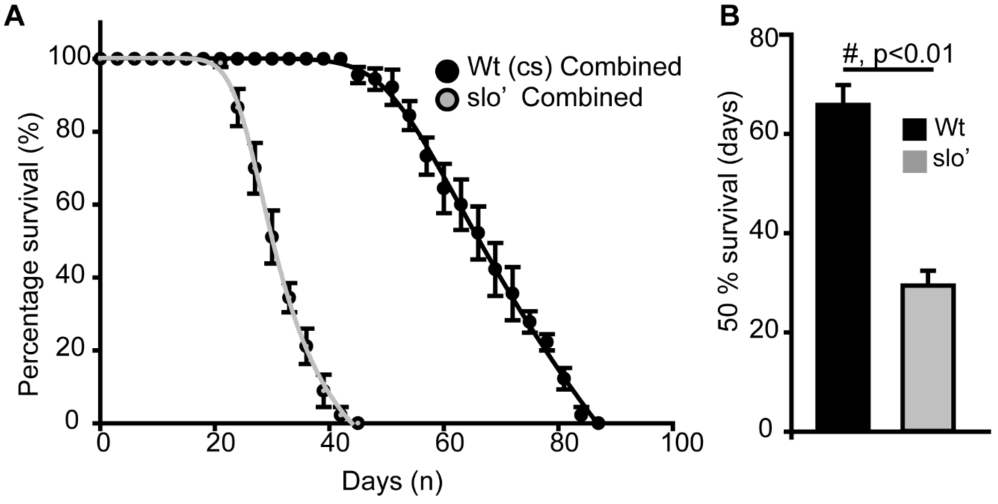

Supplementary Figure. S3

Increased levels of protein aggregates in *slo*^{1} mutants. **A**. Western blot with Anti-Poly (Ubq) antibodies indicating increased ubiquitination of proteins in *slo*^{1} mutants. Actin was used a control. **B.** Ref(2)p levels in wt and *slo*^{1} mutants lysates. Tubulin was used a control.

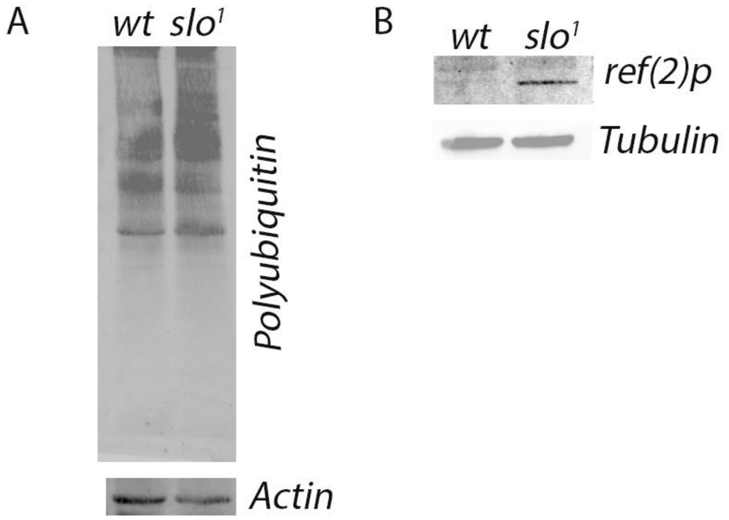

Supplementary Figure. S4

Heat map of oxidative stress-related genes altered in *slo*[1] mutants in comparison to wt flies.

The color range indicates a fold change in gene expression.

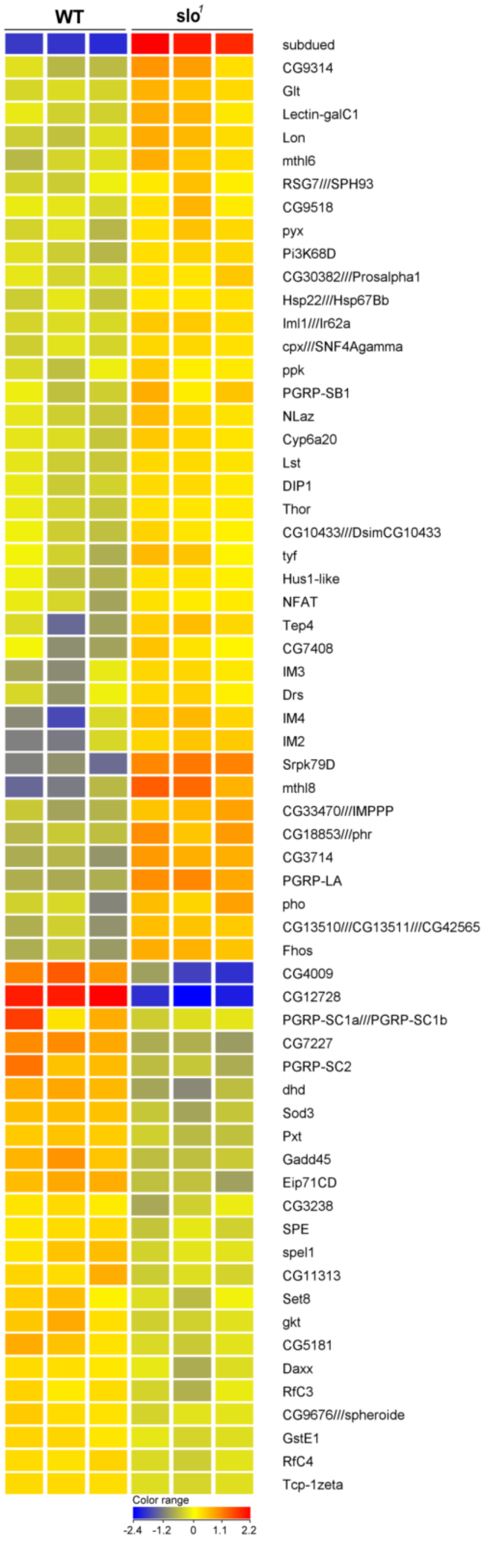

Supplementary Figure. S5

Females overexpressing human (Hs) BK_{Ca} do not show a change in life span as compared to control flies (**A**). (**B**) Bar graph showing 50% survival of control and Hs BK_{Ca} overexpressing female flies.

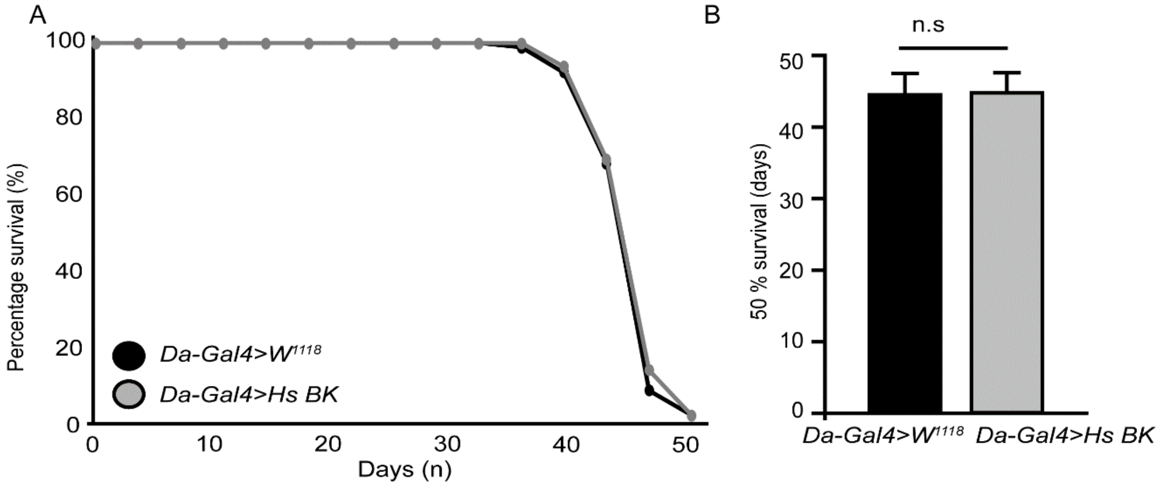

Supplement: Supplementary file 1 [file cells-08-00945-s001.pdf]
